# Teeth

**Published:** 1862-07

**Authors:** 


###  HALL'S JOURNAL OF HEALTH.

Our Legitimate Scope is almost boundless: for whatever begets pleasurable and harmless feelings, promotes Health; and whatever induces disagreeable sensations, engenders Disease.

WE AIM TO SHOW HOW DISEASE MAY BE AVOIDED, AND THAT IT IS BEST, WHEN SICKNESS COMES, TO TAKE NO MEDICINE WITHOUT CONSULTING A PHYSICIAN.

Vol. IX.] JULY, 1862. [No. 7.

### TEETH.

A STATEMENT is going the rounds of the newspapers and scissored magazines without "credit," and thus without authority, which prevents an appreciating public, too lazy to think for themselves, the pleasure of knowing to whom they are indebted for an article so universally practical, and yet so perfectly convincing and logical in its statements and conclusions. It is headed:

"A NEW TOOTH-POWDER.—Roasted rye is recommended as tooth-powder, from the fact that, in all those countries where bread made of rye is the food of the generality of the inhabitants, the latter are remarkable for the whiteness, strength, and durability of their teeth. Savoy and Landes are instances of this truth. Schrader has found 500 grammes of ashes of rye to contain 7 grammes of carbonate of lime, 9.8 ditto of magnesia, 7.2 of oxyd of iron and manganese, and 1.9 of silica, all of which substances have a favorable effect on the teeth. Rye, finely pulverized and used daily as a tooth-powder, is said to stop caries, and promptly cure the small abscesses which are often formed on the gums."

This show of scientific terms will cause the statement to be received as perfectly conclusive by that large class of readers which voraciously gulp down as true all that is put in print, thus saving themselves the trouble of thinking. Reflecting persons, however, will call in question the legitimacy of the sequence that pulverized roasted rye, daily used as a tooth-powder, will insure sound, white teeth, even if decay has already begun, because those who live on rye-bread have good teeth! It is this careless, incoherent, and illogical mixing up of the false and true, of mere theory with fact, which makes so many failures in life. Want of exactness in our knowledge is a striking characteristic of the American people. Knowledge is only practically valuable in proportion as it is exact. Hence those who "succeed in life" (with us "success" means making a large amount of money) rather blunder on a fortune than secure it by well-matured plans and operations. We might as well say that because taking water into the stomach satisfied thirst, washing the big toe would do the same thing.

It is beautifully and instructively true that the inhabitants of Savoy and Landes have strong, white, and durable teeth, but it does not necessarily follow that it is owing to the fact of their living on rye-bread. Perhaps it is because they do not eat much meat or do not drink whisky. May be it is because they do not drink much water in the south of France and in northern Italy, where wine is so abundant. Perhaps it is because they never read a daily newspaper, and thus are saved from burdening their memories and endangering their health and lives by multitudes of plausible but lying "receipts." But by a little scientific inquiry we may be able to decide with satisfactory conclusiveness whether there is any connection between eating rye-bread and having sound teeth. One familiar fact looking in that direction is, that certain soils will largely increase their yield of grain if lime is scattered over them. This proves that lime is a constituent of grains, and when a chemist analyzes them, he finds that lime *is* one of their largest constituents. If hens are fed on food which contains no lime, they will soon lay eggs without any shells, or will lay none at all; but give them lime to eat with their food, or give them food which has lime largely as a constituent, and the eggs will soon have shells on them. This would seem to show that egg-shells are mainly composed of lime, and such *is* the fact. As to the teeth, it is found that almost the entire bulk is lime. Some teeth are softer than others, and on examination it is found that they are soft because they have not the proper amount of lime in them.

Again, the "permanent" teeth of many of our children begin to decay before they are seven years old. On examination it is found that at some point, perhaps the center of the upper horizontal surface, there is a soft spot not covered with enamel, and it is because there was not lime enough in the system to complete the work of enameling the whole tooth—like some men who begin to build houses, and have to leave them unfinished for want of money to purchase materials to complete them. It would seem then that if grains are composed mainly of lime, and teeth wanting in whiteness, strength, and durability are deficient in lime, it is a rational inference that if grains were made the chief articles of food by a people, that people would have sound teeth. We feed on grains in the shape of bread. The main diet of the French is bread; and on our visit to the Louvre, in Paris, it was noticed, with considerable interest and wonder, that a young man or woman would paint until noon, then would make a hearty dinner on a loaf of bread and a bottle of wine, without leaving the apartment, and then paint on the remainder of the day. Persons were seen passing along the streets with loaves of bread on their shoulders a yard long. The fact is, the French make the best bread in the world, and on their bread and wine alone a man may live sumptuously every day. It very naturally follows then, that, as the main food of the French is made of grain, and Landes is in France, and the people of Landes have good teeth, and teeth are bad for want of lime, and grain (bread) has a great deal of lime in it, the way to give our children good teeth is to bring them up on bread. But that is taking rather a long jump, for we "fetch up all standing" against a stubborn little fact that all our children eat largely of bread, and yet dentists assure us that there are hundreds of children under ten years of age who have from one to half a dozen plugs of gold in their teeth. Is not the reader sorry to have such a beautiful theory spoiled, to say nothing of the trouble of hunting up another? But we must follow out the investigation. It never does any good to "give up," to get "discouraged," especially in matters of this kind; for this thing of having good teeth is one of prime importance in a variety of ways. It prevents dyspepsia; it adds to the pleasure and comfort of eating. Without good teeth the most splendid "figure"

is nothing, and the handsomest face is not worth looking at. What woman would knowingly and willingly wed a man without "ivories"? The smile of beauty, how enrapturing! But if that smile displays a snaggled tooth, blackened and half-decayed, what a fall!—nothing less than fathomless! Young ladies, a fine set of pearly-white teeth (of your own!) will be of greater service to you in getting a good husband, a man of fine, elevated tastes, than the stocks, bonds, and mortgages of your father.

But so far from the grain and tooth theory being spoiled, a little further investigation will add both to its beauty and truthfulness. The people of Landes eat their bread with all the lime in it. We throw the lime away and eat the remainder! Landes and Savoy are out-of-the-way countries. The inhabitants are small farmers and poor, hence are careful, waste nothing, and prepare their food in the most primitive manner. In short, they eat the whole grain, either boiling it as we do rice or cracked wheat, or pound or grind it into coarse meal for bread, thus consuming the whole grain, husk, kernel, pith, heart, and all. It is true of grains as it is of the potato, the most nutritious and wholesome part is that immediately under the outer skin. The outer eighth of an inch of the potato contains more nourishment than all the remainder; hence it is a shameful waste to peel a potato with a knife, of which many of the poor are unfortunately ignorant to their own great loss. Thus it is that the outer portion of ground grain, called the "bran," is richest in nutriment and contains nearly all the lime; but by refining it away, in our efforts to get a "fine" and "white" flour, we but eat the refuse and throw away the substance, and thus lose the lime which gives strength to the bones, durability to the teeth, and vigor to the brain, through the pure, perfect, and life-giving blood which the consumption of the whole grain makes.

In point of physical vigor and development it would be of incalculable value to our country if the children were allowed to take nothing for their breakfast and supper, as their general habit, until the twelfth year is completed, but milk with mush, cracked wheat, porridge, or other forms of food which include the entire grain. Oat-meal porridge is the main article of food in most Scotch families, and they are among the most enduring race of men. Their tenacity, their power of adherence, of "holding on," has become a proverb, not only physically but morally. Who like a Scotchman could "hold on" so bravely to his religion, in spite of the "boot"? Who like Sandy could clutch his dollar, defiant of the pirate's thumb-screw? And who but Sandy would stick to his pitcher of hot whisky-punch, albeit the ship was taking its final plunge to unfathomable depths?

In confirmation of these views, Dr. John Allen, of this city, one of the fathers of American dentistry, states, in a scientific essay on the "Development of the Natural Teeth," that seventy-one parts out of a hundred of each tooth are of lime in some form—that is, of the bony structure of the tooth; while the enamel, or vitreous substance which covers the external surface of the crown of the human tooth, contains over ninety-four per cent of lime. Dr. Allen's article has come to hand since the above was written, and it would seem as if he had prepared it exactly in the form best suited to our mode of illustration; for after showing how large a constituent of the tooth, and especially of the enamel, lime is, he proceeds to give tables showing how much lime the whole product of the grain contains, and then how little in comparison bread contains which has been deprived of its bran, for the little benefit of having a finer and whiter article of flour. Let it be remembered here that there is no specific virtue in rye beyond what is found in the other grains.

In 500 lbs. of whole grain
There are 12 lbs. of fat elements,
35 of flesh,
85 of bone.
500 lbs. of fine flour contain
10 lbs. of fat elements,
65 of flesh,
30 of bone!
500 lbs. of "bran" contain
30 of fat elements,
00 of flesh!
125 of bone!

These statements need no comment. They can not be disputed, and all argument against them is altogether useless; and all this about, not a "dog-tooth," or "sweet tooth," but a "bread-tooth;" teeth made beautiful, and white, and strong, and enduring, by eating largely of bread made of coarse flour, whether of corn, wheat, barley, oats, or rye. But as to the efficacy of tooth-powders made out of roasted rye, simply because persons who eat rye-bread have good, sound teeth, it is only the argument of an impracticable ass, or some needy, seedy dentist who wanted to "make a raise" by selling burnt rye at twenty-five cents a box, (of a table-spoonful!) But forty "fies" be on any editor who would cumber his columns with such a glaring *non sequitur*. Our Philadelphia exchanges for example!

